# Supplementary material for: Diet-Treated Gestational Diabetes Mellitus Is an Underestimated Risk Factor for Adverse Pregnancy Outcomes: A Swedish Population-Based Cohort Study
Source: Nutrients. 2022 Aug 16;14(16):3364. doi: 10.3390/nu14163364 (PMC9414969; doi:10.3390/nu14163364)
Supplement: Supplementary file 1 [file nutrients-14-03364-s001.zip › Supplementary Materials - Table S3_crude OR_neonatal outcomes.pdf]

Table S3. Neonatal outcomes (crude odds ratio) in singleton pregnancies diagnosed with diet or insulin treated GDM.

		Background Population [a] n = 1,441,338		Diet vs Background n = 8851				Insulin vs Background n = 5391				Insulin vs Diet	
		n	%	*n*	%	OR	95% CI	*n*	%	OR	95% CI	OR	95% CI
**Preterm delivery (< 37 weeks)**		70,939	4.9	713	8.1	1.70	1.57-1.83	634	11.8	2.58	2.37-2.80	1.52	1.36-1.70
**Macrosomia** [b]	**≥ 4000 g**	278,997	19.4	2216	25.0	1.39	1.33-1.46	1727	32.0	1.96	1.85-2.08	1.41	1.31-1.52
	**≥ 4500 g**	55,086	3.8	557	6.3	1.69	1.55-1.84	531	9.8	2.75	2.51-3.01	1.63	1.44-1.84
	**≥ 5000 g**	7149	0.5	114	1.3	2.62	2.17-3.15	100	1.9	3.79	3.11-4.63	1.45	1.11-1.90
**LGA-SD** [c]		49,013	3.4	911	10.3	3.26	3.04-3.49	1158	21.6	7.79	7.29-8.32	2.39	2.18-2.63
**LGA-90** [d]		139,811	9.7	1666	18.8	2.16	2.05-2.28	1748	32.4	4.47	4.22-4.73	2.07	1.91-2.24
**SGA-SD** [e]		33,544	2.3	200	2.3	0.97	0.84-1.12	89	1.7	0.71	0.57-0.87	0.73	0.57-0.94
**SGA-10** [f]		148,846	10.3	835	9.4	0.91	0.84-0.97	308	5.7	0.53	0.47-0.59	0.58	0.51-0.67
**Intrauterine death** [g]		4509	0.3	32	0.4	1.19	0.84-1.69	18	0.3	1.11	0.70-1.76	0.92	0.52-1.65
**Perinatal mortality** [h]		5925	0.4	37	0.4	1.02	0.74-1.41	24	0.5	1.09	0.73-1.63	1.07	0.65-1.75
**Apgar score < 4 at 5 min.**		4943	0.3	42	0.5	1.39	1.02-1.88	48	0.9	2.61	1.96-3.47	1.88	1.24-2.86
**Hypoglycemia**		32,274	2.2	1774	20.0	10.94	10.38-11.54	1590	29.5	18.26	17.21-19.38	1.67	1.54-1.81
**Birth trauma**		2809	0.2	42	0.5	2.44	1.80-3.31	40	0.7	3.83	2.80-5.24	1.57	1.02-2.42
**Hyperbilirubinemia**		51,522	3.6	492	5.6	1.59	1.45-1.74	408	7.6	2.21	2.00-2.44	1.39	1.21-1.59
**Respiratory distress**		38,042	2.6	303	3.4	1.31	1.17-1.47	331	6.1	2.41	2.16-2.70	1.85	1.57-2.17

GDM gestational diabetes mellitus, *N* number of individuals, *OR* odds ratio, *CI* confidence interval, *LGA* large for gestational age, *SD* standard deviation, *SGA* small for gestational age.

[a] Background population as reference; pregnancies with gestational diabetes mellitus, type one and type two diabetes mellitus excluded. [b] Macrosomia definition according to different cut-offs. [c] Birthweight two standard deviations above the mean; according to gestational age and sex. [d] Birthweight >90th percentile. [e] Birthweight two standard deviations below the mean; according to gestational age and sex. [f] Birthweight <10th percentile. [g] All malformations excluded. [h] Stillbirth or early neonatal death at ≤6 days of age, all malformations excluded.
