# Supplementary material for: Diet-Treated Gestational Diabetes Mellitus Is an Underestimated Risk Factor for Adverse Pregnancy Outcomes: A Swedish Population-Based Cohort Study
Source: Nutrients. 2022 Aug 16;14(16):3364. doi: 10.3390/nu14163364 (PMC9414969; doi:10.3390/nu14163364)
Supplement: Supplementary file 1 [file nutrients-14-03364-s001.zip › Supplementary Materials - Table S4_Descriptives insulin vs diet GDM.pdf]

Table S4. Comparison of study population characteristics for insulin vs diet treated GDM.

	Insulin vs Diet n = 5391 (Insulin)	
		p-Value [a]
Maternal characteristics:		
**Maternal age at delivery (years); mean ± SD**	32.7 ± 5.4	<0.001
**BMI at first prenatal visit (kg/m^2); mean ± SD**	30.3 ± 6.5	<0.001
**Underweight [b] ; *n* (%)**	35 (0.6)	<0.001
**Normal weight [b] ; *n* (%)**	1042 (19.3)	<0.001
**Overweight [b]; *n* (%**	1403 (26.0)	<0.073
**Obese class I [b]; *n* (%)**	1161 (21.5)	<0.001
**Obese class II-III [b] ; *n* (%)**	1088 (20.2)	<0.001
**Multipara; *n* (%)**	3750 (69.6)	<0.001
**Non-Nordic; *n* (%)**	2351 (43.6)	<0.001
**Smoking; *n* (%)**	554 (10.3)	<0.001
**Chronic hypertension; *n* (%)**	92 (1.7)	<0.004
Neonatal characteristics:		
**Male sex; *n* (%)**	2818 (52.3)	0.412

GDM gestational diabetes mellitus, *N* number of individuals, *SD* standard deviation, *BMI* body mass index.

Data are given as mean ± SD or as number of individuals and proportion N (%).

[a] *p*-Value: For continuous variables unpaired t-test (maternal age, BMI) was used and chi-square test for categorical variables (all other maternal and neonatal characteristics). [b] BMI classification according to the World Health Organization definition; underweight BMI < 18.5 kg/m^2, normal weight BMI 18.5-24.9 kg/m^2, overweight BMI 25.0-29.9 kg/m^2, obese class I BMI 30.0-34.9 kg/m^2 and obese class II-III BMI ≥ 35.0 kg/m^2.
