# Supplementary material for: Diet-Treated Gestational Diabetes Mellitus Is an Underestimated Risk Factor for Adverse Pregnancy Outcomes: A Swedish Population-Based Cohort Study
Source: Nutrients. 2022 Aug 16;14(16):3364. doi: 10.3390/nu14163364 (PMC9414969; doi:10.3390/nu14163364)
Supplement: Supplementary file 1 [file nutrients-14-03364-s001.zip › Supplementary Materials - Table S1_Abbreviations and ICD-codes.pdf]

Table S1. Abbreviations and ICD-codes

Abbreviations

BMI	body mass index kg/m^2
CS	cesarean section
OR	odds ratio
GDM	gestational diabetes mellitus
LGA	large for gestational age
SGA	small for gestational age
SMBR	Swedish medical birth registry
SD	Standard deviation
ICD	International classification of disease

**Diagnosis**	**ICD-10 codes**
GDM	O244A, O244B
Chronic hypertension	O10.0, O10.2, O10.4, O10.9
Gestational hypertension	O13.9
Preeclampsia mild	O14.0
Preeclampsia severe	O14.1A, O14.1B or O14.1X
HELLP, unspecified	O14.2, O14.9
Shoulder dystocia	O66.0
Anal sphincter injury (third and fourth degree perineal laceration)	O70.2, O70.2C, O70.2D, O70.2E, O70.2X, O70.3
IUFD	O36.4
Malformations	Q00-Q99
Neonatal hypoglycemia	P70.0, P70.1, P70.4A, P70.4B, P70.4, P70.3
Birth trauma	P11.5, P11.9, P14.0, P14.1, P14.2, P14.3, P130, P131, P100, P101, P102, P103, P104, P108 and P109
Hyperbilirubinemia	R17.9, R17.0, P59.9 and P59.0
Respiratory distress	P22.0, P22.1, P22.8, P22.9 and P28.5.
