# Supplementary material for: Diet-Treated Gestational Diabetes Mellitus Is an Underestimated Risk Factor for Adverse Pregnancy Outcomes: A Swedish Population-Based Cohort Study
Source: Nutrients. 2022 Aug 16;14(16):3364. doi: 10.3390/nu14163364 (PMC9414969; doi:10.3390/nu14163364)
Supplement: Supplementary file 1 [file nutrients-14-03364-s001.zip › Supplementary Materials - Table S2_crude OR_maternal outcomes.pdf]

Table S2. Maternal pregnancy outcomes (crude odds ratio) among women with diet or insulin treated GDM in singleton pregnancies.

	Background Population [a] *n* = 1,441,338		Diet vs Background *n* = 8851				Insulin vs Background *n* = 5391				Insulin vs Diet	
	n	%	*n*	%	OR	95% CI	*n*	%	OR	95% CI	OR	95% CI
**Gestational hypertension**	13,531	0.9	177	2.0	2.15	1.85-2.50	134	2.5	2.69	2.26-3.20	1.25	1.00-1.57
**Preeclampsia, overall**	37,383	2.6	500	5.6	2.25	2.05-2.46	428	7.9	3.24	2.93-3.58	1.44	1.26-1.65
**Preeclampsia, mild**	25,879	1.8	376	4.2	2.43	2.19-2.70	314	5.8	3.38	3.02-3.79	1.39	1.20-1.63
**Preeclampsia, severe**	10,079	0.7	106	1.2	1.72	1.42-2.09	95	1.8	2.55	2.18-3.12	1.48	1.12-1.96
**Cesarean section**	213,477	14.8	1853	20.9	1.52	1.45-1.60	1654	30.7	2.55	2.40-2.70	1.67	1.55-1.81
**Induction**	162,976	11.3	1643	18.6	1.79	1.69-1.89	1908	35.4	4.30	4.06-4.55	2.40	2.22-2.60
**Vacuum extraction**	102,528	7.1	587	6.6	0.93	0.85-1.02	314	5.8	0.81	0.72-0.91	0.87	0.76-1.00
**Shoulder dystocia**	2855	0.2	55	0.6	3.15	2.41-4.12	53	1.0	5.00	3.81-6.57	1.59	1.09-2.32
**Anal sphincter injury [b]**	5041	0.4	32	0.6	1.27	0.90-1.80	18	0.6	1.23	0.78-1.96	0.97	0.54-1.73

GDM gestational diabetes mellitus, *N* number of individuals, *OR* odds ratio, *CI* confidence interval.

[a] Background population as reference; pregnancies with gestational diabetes mellitus, type one and type two diabetes mellitus excluded. [b] All cesarean sections excluded.
